# Co-Speciation of the Ectoparasite *Gyrodactylus teuchis* (Monogenea, Platyhelminthes) and Its Salmonid Hosts

**DOI:** 10.1371/journal.pone.0127340

**Published:** 2015-06-16

**Authors:** Christoph Hahn, Steven J. Weiss, Stojmir Stojanovski, Lutz Bachmann

**Affiliations:** 1 Natural History Museum, University of Oslo, 0318, Oslo, Norway; 2 School for Biological, Biomedical and Environmental Science, University of Hull, Hull, HU6 7RX, United Kingdom; 3 Institute of Zoology, Karl-Franzens University of Graz, 8010, Graz, Austria; 4 Department of Fish Parasitology, Hydrobiological Institute, 6000, Ohrid, R. Macedonia; Biodiversity Insitute of Ontario - University of Guelph, CANADA

## Abstract

Co-speciation is a fundamental concept of evolutionary biology and intuitively appealing, yet in practice hard to demonstrate as it is often blurred by other evolutionary processes. We investigate the phylogeographic history of the monogenean ectoparasites *Gyrodactylus teuchis* and *G*. *truttae* on European salmonids of the genus *Salmo*. Mitochondrial cytochrome oxidase subunit 1 and the nuclear ribosomal internal transcribed spacer 2 were sequenced for 189 *Gyrodactylus* individuals collected from 50 localities, distributed across most major European river systems, from the Iberian- to the Balkan Peninsula. Despite both anthropogenic and naturally caused admixture of the principal host lineages among major river basins, co-phylogenetic analyses revealed significant global congruence for host and parasite phylogenies, providing firm support for co-speciation of *G*. *teuchis* and its salmonid hosts brown trout (*S*. *trutta*) and Atlantic salmon (*S*. *salar*). The major split within *G*. *teuchis*, coinciding with the initial divergence of the hosts was dated to ~1.5 My BP, using a Bayesian framework based on an indirect calibration point obtained from the host phylogeny. The presence of *G*. *teuchis* in Europe thus predates some of the major Pleistocene glaciations. In contrast, *G*. *truttae* exhibited remarkably low intraspecific genetic diversity. Given the direct life cycle and potentially high transmission potential of gyrodactylids, this finding is interpreted as indication for a recent emergence (<60 ky BP) of *G*. *truttae* via a host-switch. Our study thus suggests that instances of two fundamentally different mechanisms of speciation (co-speciation vs. host-switching) may have occurred on the same hosts in Europe within a time span of less than 1.5 My in two gyrodactylid ectoparasite species.

## Introduction

Co-evolution is a fundamental concept in evolutionary biology and in very general terms refers to “the change of a biological object triggered by the change of a related object" [1]. Co-evolution in host-parasite- or mutualistic systems is a special case defined as the reciprocal adaptive change of two antagonists through reciprocal selective pressures [2]. Another closely related phenomenon is co-speciation, whose characteristics can but need not fit the above definition of co-evolution. In host-parasite systems, co-speciation is thought to occur when the association between host and parasite species is close and speciation of the host results in parallel speciation of the parasite. The concept of co-speciation was originally proposed for avian parasites and their hosts [3,4], and the expectation that hosts and their parasites should evolve in synchrony is known as Farenholz’s rule [5]. However, host-parasite co-speciation is often difficult to demonstrate as it may be blurred by other evolutionary processes (reviewed e.g. in [6]), including (i) host switching, i.e. a parasite species partly or fully switches to another host species, (ii) duplication, i.e. parasite speciation without host speciation (sometimes also referred to as intra-host speciation [7]), and (iii) lineage sorting, i.e. the extinction or the absence of a parasite lineage in the founding population of a new host species (“missing the boat”). Parasites may also fail to speciate in response to host speciation due to increased gene flow among parasite populations relative to the host populations [8]. In a recent review [9] only 7% of all surveyed claims of co-speciation provided convincing evidence. Nonetheless a major prerequisite for tracking co-speciation events is a solid understanding of the phylogenies of the involved host and parasite species [10].

Gyrodactylid monogeneans (Platyhelminthes) are for various reasons fascinating model systems for studying the processes of speciation in host-parasite associations. The hyperdiverse genus *Gyrodactylus* currently includes about 400 ectoparasitic species [11] primarily infecting teleost fish, but this number poorly reflects the diversity of the radiation, estimated to be at least ~ 20,000 species [12]. Many species of *Gyrodactylus* have a direct life cycle and are hyperviviparous, a rare but highly efficient reproductive mode [13], which gives them the potential for rapid population growth [14]. Additionally, species of *Gyrodactylus* are capable of both sexual and asexual reproduction and new parasite populations may frequently originate from single founder individuals [14]. Given these unique reproductive features it is not surprising that host switching is believed to be the most abundant mode of speciation in *Gyrodactylus* [15]. Paradoxically, many *Gyrodactylus* species are highly host specific [12,16], a trait which is considered to result from the continuous adaptation inherent in a co-evolutionary arms race as first envisioned by Darwin [17].

However, in-depth studies on the co-evolution of gyrodactylids and their hosts are scarce. Huyse & Volckaert [18] investigated the evolutionary associations between European gobies (Gobiidae) and their *Gyrodactylus* parasites. They concluded that the radiation of highly host specific gill parasites was largely driven by host switching from the three-spined stickleback (*Gasterosteus arcuatus*) onto two gobiid genera *Pomatoschistus* and *Gobius*, followed by phylogenetically conserved host-switching among various goby hosts. In contrast, they suggested co-speciation for the less host specific fin-parasites resulting in several host-associated species complexes [18]. Another case of possible host-parasite co-speciation has been reported for the Central American guppies *Poecilia reticulate* and *P*. *picta* and their respective gyrodactylid parasites *G*. *turnbulli* and *G*. *pictae* [19].

The brown trout (*Salmo trutta*) is one of the best-studied European freshwater fish species with a complex, yet well-resolved phylogeographic structure across its wide European range. At least five distinct major lineages are distinguished for *S*. *trutta* in Europe [20–26] although a purely river-basin based phylogeographic view has been called into question in recent years [22,25,27,28]. Brown trout is an economically important species, and translocation of fish stocks has been common across large geographical distances. Only in recent years has the preservation of the genetic integrity of local brown trout populations become the objective of modern management strategies. However, while possible introgression into the gene pool of locally adapted fish populations is an important issue in conservation genetics, the effects of stocking on the integrity of host-parasite interactions, which have potentially evolved over a long period of time, have not yet been studied in detail for monogeneans. Brown trout is a suitable host for several species of *Gyrodactylus*, the three most commonly found species in Europe being *G*. *derjavinoides*, *G*. *teuchis*, and *G*. *truttae*. Parasite transmission occurs directly from host to host (no free-living stages) and detachment is usually fatal for the parasite within less than 24h (reviewed in [16]). Range expansion in gyrodactylids is thus entirely dependent on the host, and co-phylogeographic patterns might be expected.

The current study aims at identifying signatures of co-speciation in the widely distributed host-parasite system of ectoparasitic gyrodactylids and their European hosts brown trout and Atlantic salmon (*Salmo salar*). Co-phylogenetic methods are applied to evaluate phylogeographic congruence and potential allopatric co-divergence as initial steps of co-speciation.

## Materials and Methods

### Sampling

**Fig 1:**
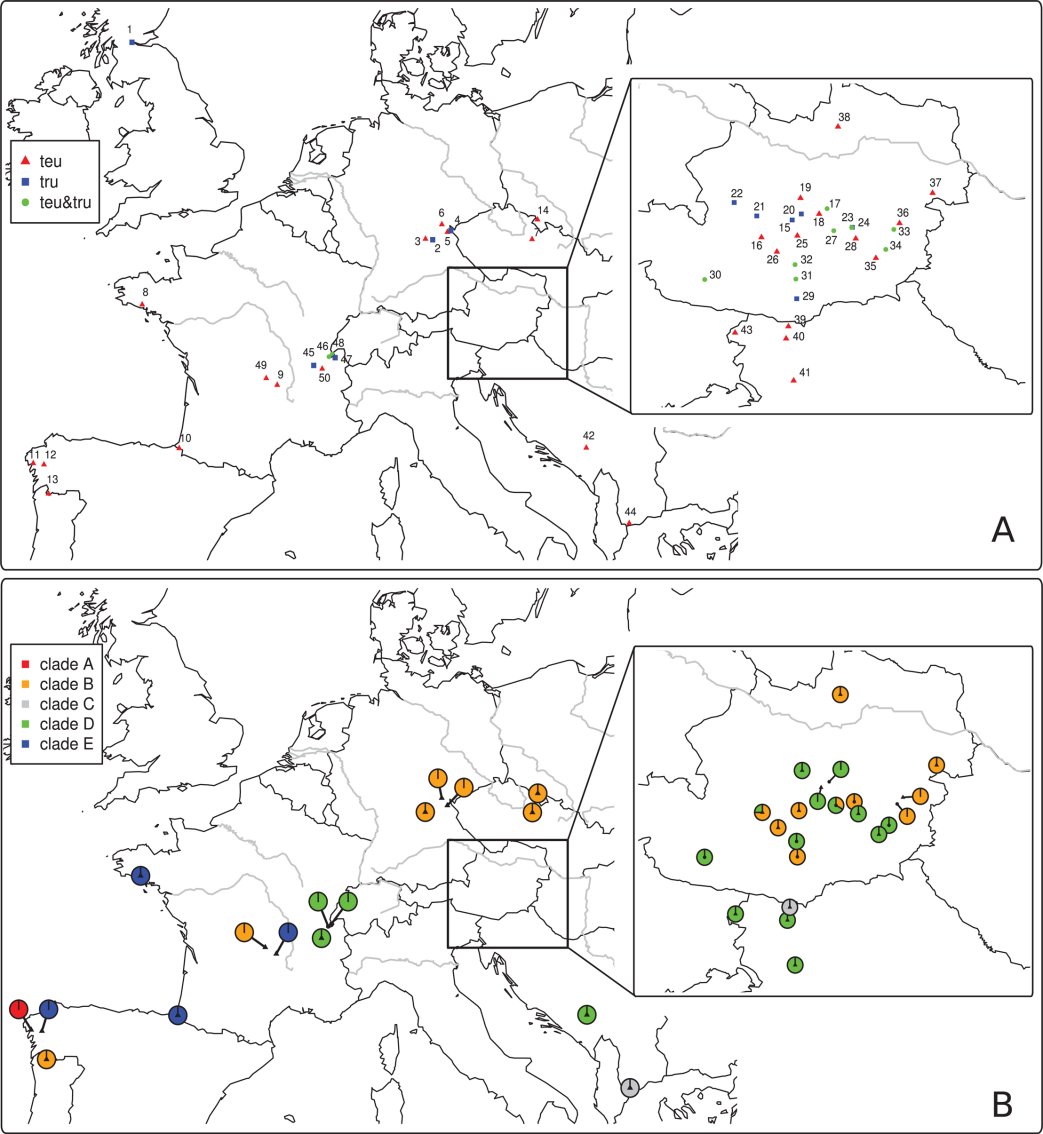
Maps illustrating (A) the principal sampling locations of the study (location numbers as in Table 1) as well as the occurrence of *G*. *teuchis* (red triangle), *G*. *trutta* (blue square), or both species (green circle) in a given location, and (B) depicting the geographic distribution of the five mtDNA clades (A-E, see Fig 2) of *G*. *teuchis* throughout the study area. The enlarged boxes represent sampling locations or mtDNA clade data in Austria and Slovenia.

*Gyrodactylus* parasites were collected from the skin and fins of ethanol preserved specimens of primarily salmonid host species: brown trout, Atlantic salmon, brook trout (*Salvelinus fontinalis*), rainbow trout (*Oncorhynchus mykiss*), and European grayling (*Thymallus thymallus*). Fish were screened for gyrodactylids using a stereomicroscope. Parasites were removed using watchmaker forceps and stored in 96% ethanol individually until further processing. No specific sampling permits were required for the collection of flatworm parasites. None of the species (fish or parasites) sampled is endangered or protected. The sampling of gyrodactylids was purely opportunistic, in that only host (fish) samples collected as part of independent routine monitoring and/or scientific collection operations were used. These operations were conducted with certified electric fishing generators, licensed operators, and with sampling permission from the local (district) level authorities, as required. *Gyrodactylus* spp. specimens were collected from 50 localities including three hatcheries (see Fig 1 and Table 1). We reiterate that the authors of this study were not responsible for, nor did they carry out fish sampling, rather they were simply given access to previously collected material.

**Table 1:** Summary of sampling locations.

river	drainage		ID	host	coordinates	date	N	
				* *			*Gteu*	*Gtru*
Bannock Burn	Forth	Atlantic	1	*St*	56.1004°N,	2006	-	1
					3.9085°W			
Aufsess	Regnitz → Main → Rhine	Atlantic	2	*St*	49.8797°N,	25.10.2011	-	2
				*Tt*	11.2293°E		-	1
Regnitz	Main → Rhine	Atlantic	3	*St*	49.9053°N,	21.10.2011	2	-
					10.8660°E			
Selb	Eger → Elbe	Atlantic	4	*St*	50.1648°N	24.10.2011	-	2
					12.1450°E			
Eger	Elbe	Atlantic	5	*St*	50.1203°N	24.10.2011	2	-
					11.9711°E			
Selbitz	Saale → Elbe	Atlantic	6	*St*	50.3605°N	24.10.2011	2	-
					11.6903°E			
Loučná	Elbe	Atlantic	7	*St*	49.8935°N,	05.12.2011	2	-
					16.2311°E			
Scorff	Blavet	Atlantic	8	*Ss*	47.8351°N,	19.09.2011	1	-
				*St*	3.3941°W		2	-
Allier	Loire	Atlantic	9	*Ss*	45.3020°N,	15.03.2011	1	-
				*St*	3.4033°E		2	-
Nivelle	-	Atlantic	10	*St*	43.3070°N,	15.09.2011	2	-
					1.5297°W			
Donas	Tambre	Atlantic	11	*St*	42.8353°N,	07.2012	2	-
					8.8752°W			
Ulla	-	Atlantic	12	*St*	42.7946°N	07.2012	2	-
					8.3362°W			
Vilamea	Caldo	Atlantic	13	*St*	41.8755°N,	07.2012	2	-
					8.1069°W			
Scinawka	Nysa Kłodzka → Oder	Baltic Sea	14	*Tt*	50.5141°N,	20.05.2011	2	-
					16.5018°E			
Palten	Enns → Danube	Black Sea	15	*St*	47.5245°N,	17.07.2009	-	10
				*Tt*	14.3887°E		-	1
Schwarzenseebach	Enns → Danube	Black Sea	16	*Sf*	47.3390°N,	16.11.2009	2	-
				*St*	13.9164°E		2	-
Hinterwildalpenbach	Enns → Danube	Black Sea	17	*St*	47.6455°N,	21.09.2009	2	1
				*Sf*	14.9239°E		-	2
Erzbach	Enns → Danube	Black Sea	18	*St*	47.5886°N,	07.10.2009	2	-
					14.8024°E			
Leerensackbach	Enns → Danube	Black Sea	19	*St*	47.7583°N,	12.09.2006	1	-
					14.5158°E			
Enns	Danube	Black Sea	20	*St*	47.5887°N,	18.10.2008	-	2
					14.528°E			
Riedlbach	Traun → Danube	Black Sea	21	*St*	47.5678°N,	28.09.2011	-	2
					13.8451°E			
Weissenbach	Traun → Danube	Black Sea	22	*St*	47.7112°N,	22.09.2009	-	2
					13.4977°E			
Mürz Hafendorf	Mur → Drau → Danube	Black Sea	23	*St*	47.4467°N,	11.10.2008	-	2
				*Tt*	15.312°E		-	1
Mürz Kapfenberg	Mur → Drau → Danube	Black Sea	24	*St*	47.445°N	06.10.2008	2	-
				*Tt*	15.3022°E		-	1
Pöls	Mur → Drau → Danube	Black Sea	25	*St*	47.3565°N,	05.10.2009	1	-
				*Sf*	14.4680°E		1	-
Katschbach	Mur → Drau → Danube	Black Sea	26	*Sf*	47.1853°N	23.07.2009	2	-
				*St*	14.1537°E		8	-
Vordernbergerbach	Mur → Drau → Danube	Black Sea	27	*St*	47.4100°N,	23.07.2009	2	1
				*Om*	15.0264°E		1	-
Mur Mixnitz	Drau → Danube	Black Sea	28	*St*	47.3258°N	03.10.2009	2	-
					15.3642°E			
Gurk	Drau → Danube	Black Sea	29	*Tt*	46.6848°N,	21.10.2008	-	2
					14.4577°E			
Möll	Drau → Danube	Black Sea	30	*St*	46.8895°N,	27.11.2008	2	2
				*Tt*	13.0455°E		-	1
Metnitz	Gurk → Drau → Danube	Black Sea	31	*St*	46.8942°N,	17.11.2009	2	1
					14.4447°E			
Olsa	Gurk → Drau → Danube	Black Sea	32	*St*	47.0481°N,	23.09.2009	5	10
					14.4321°E			
Lafnitz	Raab → Danube	Black Sea	33	*Tt*	47.4237°N,	17.10.2008	1	1
					15.9487°E			
Feistritz	Raab → Danube	Black Sea	34	*St*	47.2105°N,	08.10.2008	2	2
				*Tt*	15.8255°E		-	1
Kötschmanngrabenbach	Raab → Danube	Black Sea	35	*St*	47.1181°N,	22.09.2011	2	-
					15.6731°E			
Pinka	Raab → Danube	Black Sea	36	*St*	47.4894°N,	08.09.2011	2	-
					16.0361°E			
Wulka	Neusiedlersee	-	37	*St*	47.8118°N,	15.10.2009	10	-
					16.5437°E			
Kleiner Kamp	Kamp → Danube	Black Sea	38	*St*	48.5192°N,	20.11.2008	10	-
					15.0922°E			
Bistrica	Sava → Danube	Black Sea	39	*St*	46.3893°N,	15.06.2011	2	-
					14.3304°E			
Besnica	Sava → Danube	Black Sea	40	*St*	46.2594°N,	15.10.2011	2	-
					14.2949°E			
Mahnečica	Sava → Danube	Black Sea	41	*St*	45.8106°N,	21.10.2011	2	-
					14.4107°E			
Tara	Sava → Danube	Black Sea	42	*St*	43.3231°N,	05.06.2010	3	-
					18.9610°E			
Kranska	Lake Prespa → Drin	Adriatic	43	*Stp*	40.9465°N,	27.10.2010	2	-
					21.1219°E			
Gljun	Soča	Adriatic	44	*Stm*	46.3217°N,	01.12.2011	10	-
					13.5116°E			
Ain	Rhone	Mediterranean	45	*St*	45.9196°N,	16.03.2011	-	2
					5.2418°E			
Allondon	Rhone	Mediterranean	46	*St*	46.1934°N,	28.09.2010	7	10
					6.0029°E			
Foron de Fillinge	Arve → Rhone	Mediterranean	47	*St*	46.1638°N,	15.06.2010	-	1
				*Tt*	6.3206°E		-	1
Versoix	Rhone	Mediterranean	48	*St*	46.2763°N,	08.10.2010	1	2
				*Tt*	6.1672°E		1	-
hatchery	Loire	Atlantic	49	*St*	-	15.03.2011	2	-
hatchery	Rhone	Mediterranean	50	*St*	-	16.03.2011	2	-
fish farm^1^	Vistula	Balitc	51	*St*	-	27.03.2002	-	?

Shown are the river names, the associated drainage, an ID number that corresponds to sites shown in Fig 1, the host species (*Om—O*. *mykiss*, *Sf—S*. *fontinalis*, *Ss—S*. *salar*, *St*—*S*. *trutta*, *Tt—T*. *thymallus*), the sites coordinates, collection date, and the number of individuals (*N*) of the two study species, *G*. *teuchis* and *G*. *truttae*, analysed at each site.

^1^Genbank accession EU304826

### Molecular methods

Total genomic DNA was extracted from individual worms as described earlier [29]. Only one parasite specimen per fish was processed to minimize the likelihood of analysing directly related *Gyrodactylus* individuals. The internal transcribed spacer 2 (ITS2) of the nuclear ribosomal gene cluster was amplified and sequenced as described previously [29] using published primers ITS4.5 (5'-CATCGGTCTCTCGAACG-3') and ITS2 (5'-TCCTCCGCTTAGTGATA-3') [30]. Obtained ribosomal sequences were subjected to a BLAST search [31] against GenBank for species identification. For the amplification of a ~1,350 bp fragment of the mitochondrial cytochrome oxidase I gene (COI) we used the forward primer teutruforw (5'-TTGTTTTCAAAACAAAAAGTGC-3') with the reverse primer teurev (5'-TACTACTTTCGTTTCATAGCCC-3') or trurev (5'-TACTTCTTTCACTCCATAACCC-3'), newly designed primer pairs for species-specific amplification in *G*. *teuchis* or *G*. *truttae*, respectively. The COI fragments were likewise amplified under standard PCR conditions as previously described [29] and sequenced (both strands) using the PCR primers and BigDye chemistry (Applied Biosystems). Sequences were quality checked and edited manually using the Sequencher 4.1.4 software (Gene Codes Corporation).

In addition to the novel COI sequences of *G*. *teuchis* and *G*. *truttae* generated in this study sequences for *G*. *derjavinoides* (EU293891), *G*. *lavareti* (EF446765), *G*. *salaris* (DQ988931), *G*. *thymalli* (EF527269), and *G*. *truttae* (EU304826) were retrieved from GenBank and included in subsequent analyses. Additionally, a dataset from previously published sequences of the mitochondrial control region (CR) of the salmonid hosts Atlantic salmon and brown trout (see Table 2) was compiled. The control region was chosen due to the extensive number of available sequences and a reasonably acceptable calibrated divergence rate in salmonid fishes. Brown trout sequences from five major geographical mtDNA lineages (Adriatic, Atlantic, Danubian, Duero, Mediterranean) were carefully selected to capture a wide range of the reported European diversity. Alignments were generated using MAFFT [32] and for parasite sequences manually edited and analyzed (p-distances, dS/dN ratio) in MEGA5 [33] and DNAsp [34].

**Table 2:** General information on the host sequences used in the current study.

species	GenBank	mtDNA lineage	mt haplotype
*St*	AY185571^1^	Danube	Da3
*St*	AY185572	Danube	Da9
*St*	AY185575	Danube	Da23b
*St*	AF273086^1^	Atlantic	H1
*St*	AF273087	Atlantic	H2
*St*	EF530495	Atlantic	ATcs33
*St*	EF530513^1^	Duero	DUcs1
*St*	EF530514	Duero	DUcs2
*St*	EF530515	Duero	DUcs3
*St*	AY836350^1^	Mediterranean	MEcs1
*St*	AY836351	Mediterranean	MEcs2
*St*	AY836352	Mediterranean	MEcs3
*St*	AY836330^1^	Adriatic	ADcs1
*St*	AY836331	Adriatic	ADcs2
*St*	AY836332	Adriatic	ADcs3
*Ss*	U12143^1^	-	-
*Ss*	JQ390056	-	-
*Ss*	AF133701	-	-

*St—S*. *trutta*, *Ss—S*. *salar*. The mtDNA haplotypes refer to the haplotype names given in the initial publications.

^1^ sequence used in host-parasite association analyses

### Phylogenetic analyses and species delimitation

The best fitting substitution models were determined using MrModeltest2 [35]. Phylogenetic relationships were inferred using Bayesian inference (BI) with MrBayes 3.2.1 [36] as well as Maximum likelihood (ML) with RAxML 7.8.3 [37]. On each alignment BI was run for four analyses with four Metropolis-coupled Markov chain Monte Carlo (MCMC) chains with an incremental heating temperature of 0.1 for 50,000,000 generations and sampled every 1,000 generations. The first 25% of the generations were discarded as “burn-in”, and posterior probabilities were estimated for the remaining generations. ML tree robustness was assessed by >100 bootstrap pseudo replicates. Molecular species delimitation was subsequently performed on the ML trees inferred from the COI sequences using the recently proposed Poisson Tree Processes (PTP) model [38] as implemented on the PTP-webserver (http://species.h-its.org/ptp/, last accessed February, 2015).

### Co-phylogenetic analyses of *G*. *teuchis* and *Salmo* spp.

**Fig 2:**
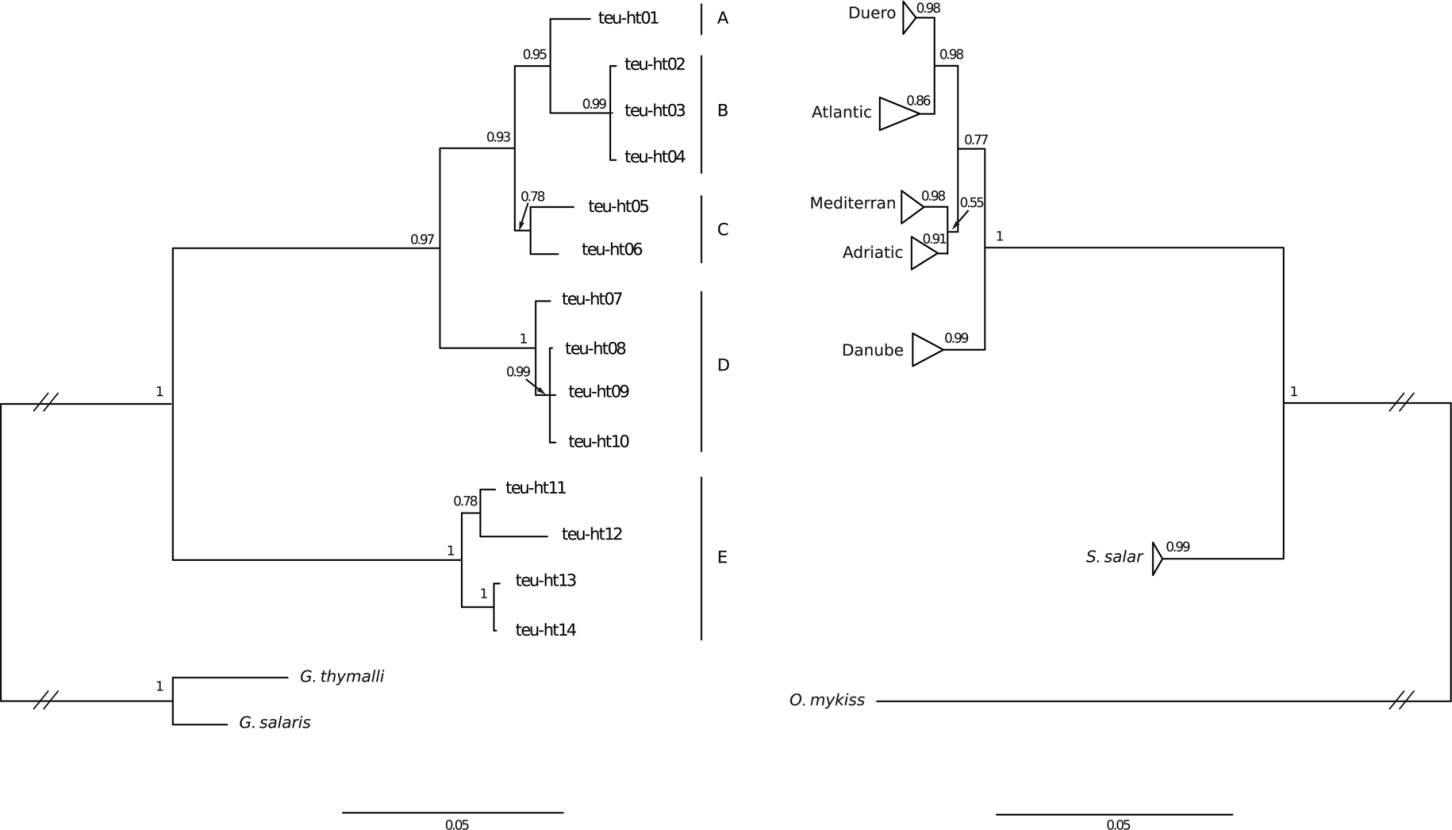
Results of Bayesian phylogenetic inference (GTR+Γ) for *G*. *teuchis* (tree on the left) based on COI gene, and for *Salmo* sp. (tree on the right) based on mitochondrial control region. The labels in the upper part of the *Salmo* tree refer to the five major lineages of *S*. *trutta*.

**Fig 3:**
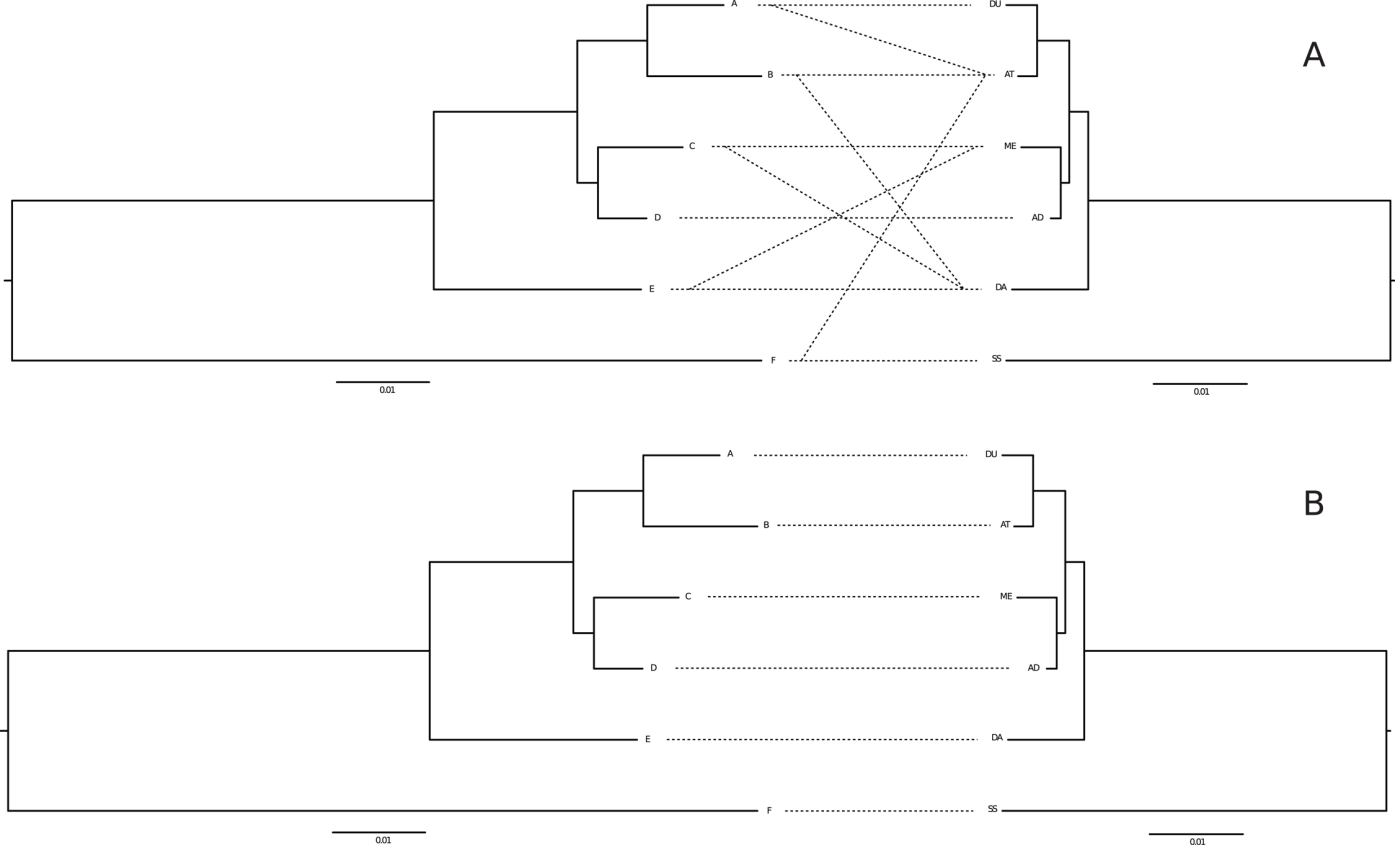
Two alternative host parasite associations between *G*. *teuchis* (left) and *Salmo* sp. (right), whereby (A) represents the HPM-1 model allowing for some ambiguity due to shared clades within drainages, and B is the most parsimonious model based on the most frequent or presumed native lineages (see methods for details). *G*. *teuchis* lineages A-E refer to the major lineages recovered in the Bayesian analysis illustrated in Fig 2. The major lineages for *Salmo* sp. are abbreviated as follows: DU—Duero, AT—Atlantic, AD—Adriatic, DA—Danube, and SS—*S*. *salar*.

To assess topological congruence of phylogenetic trees and to test for signatures of co-divergence between *G*. *teuchis* and *Salmo* spp. the programs ParaFit [39] and PACo [40] were used. Prior to analyses both the parasite and the host datasets were reduced to retain only one representative sequence for each of the major lineages. For *G*. *teuchis* five lineages were defined according to the least conservative species delimitation result (see Fig 2 and results section below). For lineages comprising more than one haplotype the most abundant haplotype was chosen as representative. For *Salmo* spp. we randomly chose one of the previously published haplotypes for each of the relevant mtDNA trout lineages (see Table 2) and one haplotype for *Salmo salar*. Subsequently, two host-parasite association matrices (HPM) were constructed to evaluate geographical congruence of parasite and host lineages. HPM-1 (see Fig 3A) allowed for ambiguity in the geographical associations between host and parasite lineages. That is, parasite haplotypes could be assigned to more than one host lineage, thus taking into consideration that parasite haplotypes were detected in more than one distinct river basin, associated with major host lineages, or that parasite haplotypes originated from regions of known admixture between major host lineages.

HPM-2 (see Fig 3B), in contrast, is based on the most parsimonious host association for each parasite lineage. Here, meta-data on the sampling locations, e.g., information about earlier introduction of non-native fish stocks, were taken into consideration.

For example, *G*. *teuchis* haplotypes assigned to clade D were identified in both the Danubian and Adriatic drainages (see results section below), which harbour distinct Danubian (DA) and Adriatic (AD) trout lineages, respectively. Thus clade D is connected to lineage DA and AD in HPM-1. However, the Adriatic river in question (Soca drainage) is known to have been heavily stocked with Danubian trout (see Discussion below) and HPM-2 thus only takes into account the most parsimonious host-parasite association of *G*. *teuchis* clade D to trout lineage DA.

PACo and ParaFit tests for each HPM were conducted in R [41] and run for 100,000 permutations based on pairwise genetic (TN93 evolutionary model of substitution) and patristic distances (computed from ML trees inferred with MEGA5 [33]), respectively.

### Estimating the nucleotide substitution rate for the mitochondrial COI gene in *G*. *teuchis*

The flatworm fossil record is rather poor and hence direct calibration points are scarce [42]. Based on topological congruence between the molecular phylogenies of *G*. *teuchis* and its hosts *S*. *truttae* and *S*. *salar* the nucleotide substitution rate for the COI sequences of *G*. *teuchis* was inferred using an indirect calibration, i.e. the previously inferred divergence time of approx. 0.6 My for the Atlantic and Danubian lineages of *S*. *trutta* (see e.g. [20,25]). Accordingly the tMRCA prior for the corresponding node in *G*. *teuchis* was imposed as a lognormal distribution with a mean of 0.6 My (SD = 0.1 My). We applied a speciation Yule tree prior and the defaults of BEAUTi v. 1.8.0 [43] for all other parameters. Initial BEAST runs using a relaxed molecular clock with uncorrelated lognormal distributed substitution rates revealed a rate variation with a standard deviation close to zero, which indicates that a clock-like mode of substitution cannot be rejected. Consequently, all analyses were repeated under a strict molecular clock model with otherwise identical settings. To compare the inferred node ages between host and parasite the same procedure was applied to the host dataset. For each analysis, three independent Markov chains were run for 20 million generations and parameters were sampled every 1,000 generations. Parameter estimates of the independent analyses were checked for convergence using Tracer v. 1.5 [44] and combined with LogCombiner v. 1.8.0 [43].

## Results

A total of 39 of 50 sampled salmonid populations (78%) were infected with *G*. *teuchis*, while *G*. *truttae* occurred in 22 populations (42%) (Table 1). Co-infection with both species was observed in 10 populations (20%). *G*. *teuchis* was thus significantly more prevalent than *G*. *truttae* (p < 0.001, Fisher’s exact test). The 189 *Gyrodactylus* individuals (n_teu_ = 122; n_tru_ = 67) analysed in the current study were collected from five salmonid host species: *Salmo trutta* (n_teu_ = 110; n_tru_ = 55), *Salmo salar* (n_teu_ = 2), *Salvelinus fontinalis* (n_teu_ = 5; n_tru_ = 2), *Oncorhynchus mykiss* (n_teu_ = 1), *Thymallus thymallus* (n_teu_ = 4; n_tru_ = 10). In a few brown trout and grayling populations, we also detected *G*. *derjavinoides* and *G*. *thymalli*, respectively.

### *Gyrodactylus truttae*

**Fig 4:**
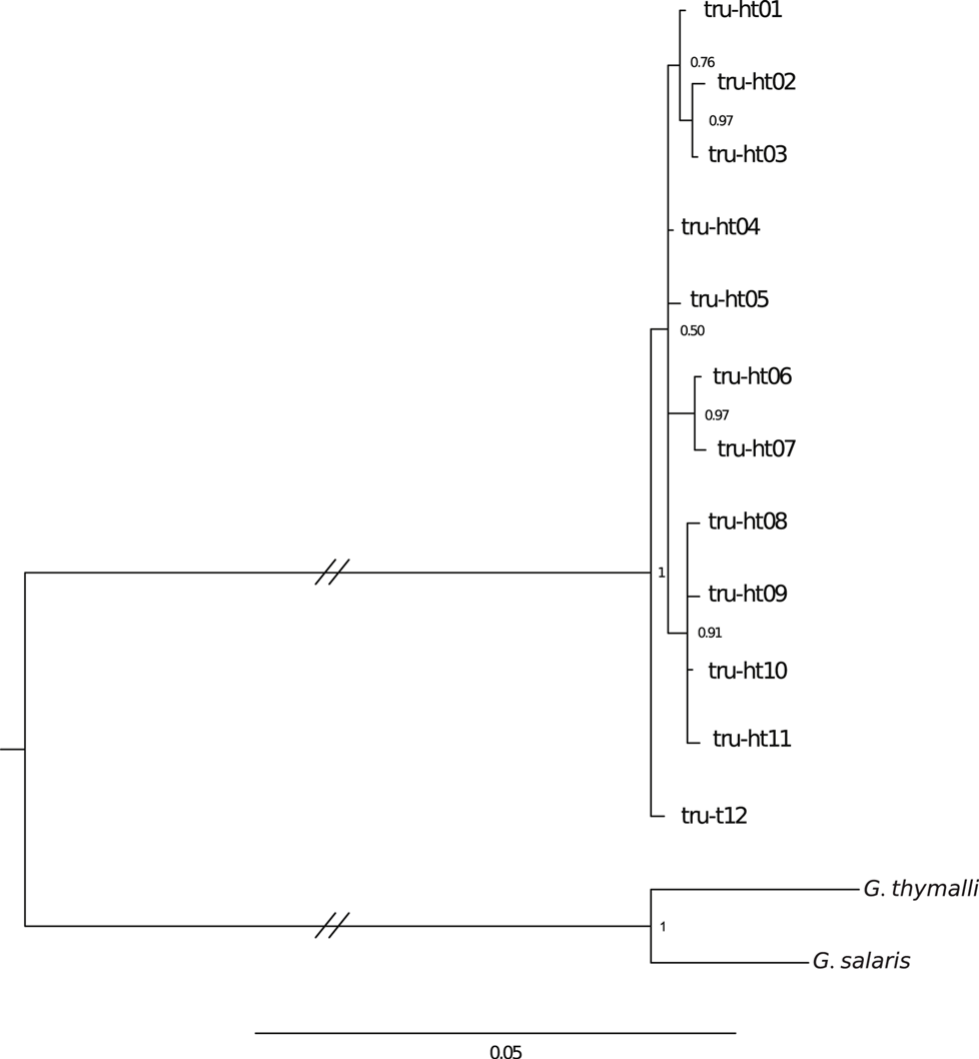
Bayesian inference (GTR+Γ) of the intraspecific phylogenetic relationships within *G*. *truttae* based on COI.

In total, we detected 11 novel mitochondrial haplotypes across the 67 analyzed *G*. *truttae* specimens from the 22 infected populations. None were identical to the previously published sequence of *G*. *truttae* from a Polish fish farm (GenBank entry EU304826). Nucleotide sequences of the new haplotypes were submitted to GenBank (accessions: KR080729—KR080739). The final alignment of 1,349 bp contained 14 variable sites (dS/dN ratio = 12/2), of which seven were parsimony informative. The average GC-content was 35.7%, and the transition/transversion ratio was 4.02. The average K2P-distance between any two COI haplotypes was 0.003 (range 0.001–0.005). Bayesian inference and ML methods recovered identical tree topologies. Automated species delimitation based on the PTP model assigned all *G*. *truttae* haplotypes to one single species (p<0.001). Table 3 summarizes the geographic distribution of individual haplotypes and Fig 4 presents the phylogeographic hypothesis for the species. Additionally, the ITS2 region was sequenced in 16 individuals from 14 sampling sites (see Table 3). BLAST search of the ITS2 sequences in all cases returned *G*. *truttae* as the best hit with > 99% identity. Only one variable site was detected at ITS2 position 86, which resulted in three variants (see Table 3). Variant A was identical to previously published sequences for *G*. *truttae* and was found in 11 populations (Table 3) and on two host species (*S*. *trutta*, *T*. *thymallus*). Variant B was found in two localities and two host species (*S*. *trutta*, *T*. *thymallus*). Variant C was only detected on *S*. *trutta* at one single site. No obvious congruence in relationships between COI haplotypes and ITS2 sequences could be identified. The novel ITS2 sequences obtained in the current study were submitted to GenBank (accessions: KR080740—KR080742).

**Table 3:** Geographical distribution of mitochondrial haplotypes and ITS sequences for *G*. *truttae*.

ID	host	COI haplotypes												ITS2 variant
		1	2	3	4	5	6	7	8	9	10	11	12	
1	*St*												1	-
2	*St*	2												A
2	*Tt*		1											-
4	*St*												2	-
15	*St*	10												A
15	*Tt*										1			-
17	*Sf*	2												-
17	*St*	1												-
20	*St*						1		1					B
21	*St*	2												-
22	*St*	2												-
23	*St*									2				A
23	*Tt*									1				-
24	*Tt*	1												A
27	*St*	1												-
29	*Tt*						2							A
30	*Tt*	1												A
30	*St*							2						A
31	*St*					1								A
32	*St*	4							6					A
33	*Tt*	1												-
34	*Tt*								1					-
34	*St*								2					A
45	*St*	2												-
46	*St*	3										7		C
47	*Tt*	1												B
47	*St*				1									B
48	*St*											2		A
51	*St*			?										A

Shown is the location ID (as shown in Table 1), the host species (*Sf—S*. *fontinalis*, *Ss—S*. *salar*, *St*—*S*. *trutta*, *Tt—T*. *thymallus*) and numbered COI and ITS2 variants (described further in Table 4).

### *Gyrodactylus teuchis*

Among the 122 analyzed *G*. *teuchis* individuals we identified 14 novel mitochondrial haplotypes (GenBank accessions: KR080710—KR080723). The final alignment of 1,355 bp contained 187 variable sites (dS/dN ratio = 196/9) of which 160 were parsimony informative. The average GC-content was 36.3%, and the transition/transversion ratio was 5.98. The average K2P-distance between any two COI haplotypes was 0.061 (range 0.001–0.11). BI and ML methods yielded identical tree topologies (Fig 2). The PTP model delimited (p < 0.001) a total of two species in the *G*. *teuchis* dataset when using *G*. *salaris* and *G*. *thymalli* as outgroups (species 1—ht01-10; species 2—ht11-14). However, when using *G*. *lavareti* (GenBank EF446765) as an outgroup five species were delimited (species 1—ht01; species 2—ht02-ht-4; species 3—ht05-ht06; species 4—ht07-ht10; species 5—ht11-14). The genetic divergence estimates between the two outgroup species and *G*. *teuchis* were similar (*G*. *lavareti* vs. *G*. *teuchis* K2P = 0.186; *G*.*salaris*/*thymalli* vs. *G*. *teuchis* K2P = 0.218). Sequencing of the ITS2 region in 24 *G*. *teuchis* individuals from 22 sampling localities revealed five distinct ITS2 variants (see Table 4 and Table 5). Variant A was identical to the ITS2 sequence previously reported in the re-descriptions of *G*. *teuchis* [29,45] and was detected at 18 localities in parasites isolated from four host species (*S*. *fontinalis*, *S*. *salar*, *S*. *trutta*, *T*. *thymallus*), while variant D was found at two locations in parasites from two host species (*S*. *fontinalis*, *S*. *trutta*). In contrast, variants B, C, and E were each found at only one location in parasites from *S*. *trutta* (see Table 5). Overall there were seven variable sites, none of which were parsimony informative (Table 4). BLAST searches of the ITS2 sequences always returned *G*. *teuchis* as the best match with more than 99% identity. The novel ITS2 sequences obtained in this study were submitted to GenBank (accessions: KR080724—KR080728).

**Table 4:** Summary of ITS2 variants observed in *G*. *teuchis* and *G*. *truttae*.

*G*. *teuchis*	ITS2 position							*G*. *truttae*	ITS2 position
variant	58	68	100	127	173	180	255	variant	86
A	A	T	T	G	A	G	C	A	A
B	G	A	C	R	A	G	C	B	T
C	R	W	Y	R	A	G	C	C	W
D	R	T	Y	G	R	R	Y		
E	A	T	T	G	R	R	Y		

The geographical distribution of the variants is shown in Table 3 and Table 5 for *G*. *truttae* and *G*. *teuchis*, respectively.

**Table 5:** Geographical distribution of mitochondrial haplotypes and ITS sequences for *G*. *teuchis*.

ID	host	COI haplotype														ITS2
		A	B			C		D				E				variant
		1	2	3	4	5	6	7	8	9	10	11	12	13	14	
3	*St*			2												-
5	*St*			2												-
6	*St*			2												-
7	*St*			2												-
8	*Ss*											1				A
8	*St*											2				A
9	*Ss*											1				A
9	*St*											2				A
49	*St*			2												-
10	*St*													1	1	B
11	*St*	2														C
12	*St*												2			A
13	*St*				2											A
14	*Tt*			2												A
16	*Sf*			1				1								D
16	*St*			2												E
17	*St*								2							-
18	*St*								2							D
19	*St*								1							A
24	*St*			2												A
25	*St*		1													-
25	*Sf*			1												-
26	*Sf*			2												A
26	*St*			8												-
27	*St*			1				1								-
27	*Om*							1								-
28	*St*								2							-
30	*St*								2							A
31	*St*			2												A
32	*St*								5							-
33	*Tt*			1												-
34	*St*								2							A
35	*St*								2							-
36	*St*			2												-
37	*St*			10												A
38	*St*			10												-
39	*St*					2										-
40	*St*									2						-
41	*St*										2					-
42	*St*								3							A
43	*St*						10									A
44	*St*								2							A
46	*St*								7							A
48	*St*								1							A
48	*Tt*								1							-
50	*St*								2							-

Shown is the basin, the location ID (as shown in Table 1), the host species (*Om—O*. *mykiss*, *Sf—S*. *fontinalis*, *Ss—S*. *salar*, *St*—*S*. *trutta*, *Tt—T*. *thymallus*) and numbered COI haplotypes (letters indicate clades as in Fig 2) and ITS2 variants (described further in Table 4).

### Co-phylogenetic analyses

The phylogenetic tree based on mitochondrial COI haplotypes of *G*. *teuchis* was compared with the phylogenetic tree of brown trout and Atlantic salmon based on sequences of the mitochondrial control region (see Fig 2). At first glance the topologies of both trees appear congruent. The two alternative host-parasite associations (HPM-1 and HPM-2) tested using co-phylogenetic methods are illustrated in Fig 3. For the HPM-1 association model, global congruence between host and parasite tree topologies was significant or of borderline significance (0.06) in three of the four tests using pairwise genetic (P_PACo_ = 0.06; P_ParaFit_ = 0.089) or patristic distances (P_PACo_ = 0.04; P_ParaFit_ = 0.061). However, more convincing significant global congruence was found for HPM-2 model using both genetic (P_PACo_ = 0.035; P_ParaFit_ = 0.046) and patristic distances (P_PACo_ = 0.019; P_ParaFit_ = 0.039).

### The nucleotide substitution rate for the mitochondrial COI gene in *G*. *teuchis*

Applying the divergence time of ~ 0.6 My between the Atlantic and Danubian trout lineages [20,25] as an indirect calibration for the corresponding node in the *G*. *teuchis* phylogeny of mitochondrial haplotypes, BEAST inferred a mean substitution rate of 5.1% (95% HPD 2.9–7.7%) per million years using a strict molecular clock, which corresponds to an age of 1.46 My (95% HPD 0.8–2.2) for the most recent common ancestor of *G*. *teuchis*. The 95% confidence intervals for the inferred mean node ages for *G*. *teuchis* and its hosts overlapped in all cases. However, the mean age of nodes for *G*. *teuchis* tended to be lower than for the host trees.

## Discussion

Despite both anthropogenic and naturally caused admixture of major host lineages among major river basins, the most parsimonious host-parasite association model revealed significant global congruence in tree topologies, providing support of co-speciation of *G*. *teuchis* and its salmonid hosts (*S*. *trutta* and *S*. *salar*). The anthropogenic introduction of brown trout stocks from various Atlantic basin sources into the Danube basin is well documented, but strong evidence has also been presented for a natural postglacial north-south range expansion [22,23,25]. Recently, the genetic distinction of anthropogenically and naturally expanding Atlantic lineages of brown trout into the Danube basin has been analysed in detail, and natural admixture is at least regionally dominant over human-mediated expansion [28]. This postulated unidirectional expansion of the host *S*. *trutta* is congruent with our parasite data, reflected in the occurrence of the putative Atlantic haplotype teu-ht03 in both Atlantic and Danubian watercourses, while haplotypes associated with Danubian brown trout lineage (teu-ht07-10) were absent from rivers of the Atlantic basin. The apparently stable co-occurrence of Atlantic and Danubian *G*. *teuchis* haplotypes in some watercourses of the Austrian Danube (e.g. Schwarzenseebach, Vordernbergerbach) is likewise congruent with gradual secondary postglacial contact rather than invasion of Atlantic haplotypes due to human mediated translocation. The absence of Danubian haplotypes in some Austrian rivers most likely reflects the limited sampling. However, local replacement of Danubian haplotypes via introgression and/or subsequent selective sweeps cannot be ruled out. In contrast, the occurrence of teu-ht08 in Rhone tributaries is most likely the result of human mediated translocation of parasites along with their fish hosts as this putative Danubian haplotype was also detected in a local Rhone basin fish farm. Currently only two trout populations have been sampled for *Gyrodactylus* in the Rhone catchment and it is expected that more extensive sampling is likely to also reveal distinct Rhone haplotypes of *G*. *teuchis* corresponding to the Rhone trout lineage. The detection of the common Danubian haplotype teu-ht08 in the Adriatic Soca system is easily explained by extensive stocking of Danubian trout in Soca tributaries (A. Snoj personal communication). The trout population of the river Kranska (sometimes referred to as *S*. *peristericus* [46]) was recently genotyped [47], and the authors reported that the population is fixed for a specific Adriatic trout haplotype. There is therefore little doubt that the *G*. *teuchis* haplotype collected from this very population (teu-ht06) indeed represents a novel Adriatic haplotype, an interpretation that is also supported by co-phylogenetic analyses. The Balkans are believed to be a centre of diversity for the genus *Salmo* [46], and an extensive exploration of the associated *Gyrodactylus* species is likely to reveal further cryptic diversity. Although found in a Danube tributary, haplotype teu-ht05 was assigned to the Adriatic lineage based on the results of the PTP species delimitation. Taking into consideration the topological congruence of the host and parasite trees as illustrated in Fig 2 one might expect an association of teu-ht05 with the Mediterranean trout lineage. There have been no reports of introgression of Adriatic or Mediterranean trout into the Bistrica trout population (A. Snoj personal communication). However, it should be kept in mind that the reproductive strategy of gyrodactylids facilitates rapid population growth and population expansion starting from a very low number of founder individuals.

The *G*. *teuchis* haplotype teu-ht01 was sufficiently divergent from the remaining Atlantic haplotypes (teu-ht02-04) to be delimited as separate species by the PTP algorithm (using *G*. *lavareti* as an outgroup) and topological congruence with the *Salmo* tree indicates an association with the Duero trout lineage. We emphasize that this choice of outgroup and application of the PTP algorithm alone does not provide a definitive answer of species delineation. Rather, we apply it as an objective approach for at least delineating clades or generating hypotheses concerning species diversity. The respective sampling locality for teu-ht01 (Donas) is not part of the Duero system and the local trout population has never been genotyped. Individual trout carrying Duero clade haplotypes, however, are not limited to the Duero system [24] and have been reported to occur in the relatively nearby north-flowing Mandeo River system [48]. In the absence of a reference haplotype from an unambiguously identified Duero trout location we speculate that teu-ht01 represents a parasite lineage specific to, or at least originating with the Duero trout lineage.

Co-phylogenetic analyses clearly indicate an association between Atlantic salmon and the highly divergent clade E of *G*. *teuchis*. The haplotypes within this clade (teu-ht11-14) were collected exclusively from rivers that support sympatric populations of brown trout and Atlantic salmon. Although restocking programs aiming to restore the dwindling salmon populations in these rivers have been carried out, the native salmon populations have not gone extinct [49,50], and the results of the co-phylogenetic analyses corroborate an ancient association of *G*. *teuchis* with Atlantic salmon in these rivers. In contrast, the contemporary salmon population of Tambre River was shown to be the result of stray individuals from neighbouring rivers [51], and the traces of the ancient association of *G*. *teuchis* with salmon in this river might have disappeared with the local extinction of the native salmon stock. The observed *G*. *teuchis* haplotype, associated with the Atlantic mtDNA lineage of brown trout, may reflect a secondary occupation of an open ecological niche.

Two recent studies attempted to date various splits within Salmonidae but did not specifically address the splits within the genus *Salmo* [52,53]. Nevertheless, the divergence time between the Atlantic and Danubian mtDNA brown trout lineages of approx. 0.6 My [20,25] is largely accepted. Using this calibration point as a proxy for the split of the respective mitochondrial lineages of *G*. *teuchis* yielded a nucleotide substitution rate of 5.1% (2.9–7.7%) per million years. This estimate is substantially lower than the 13.7–20.3% per million years reported for the COI genes of *G*. *salaris* and *G*. *thymalli* [54]. When comparing both estimates it is important to note that the latter was also derived indirectly. The authors interpreted the phylogeographic pattern of the COI haplogroups recovered for *G*. *salaris* and *G*. *thymalli* as indicative of simultaneous isolation of mitochondrial lineages and the separation of the hosts into four refugia situated around the Continental Ice Cap during the beginning of the last major glaciation cycle approx. 130,000 years ago [54]. While some key parameters such as the dS/dN ratio are very similar for the data presented here and that from [54] (21.7:1 for *G*. *teuchis* and 17:1 for *G*. *salaris* and *G*. *thymalli*) other characteristics such as the GC-content (36.3% for *G*. *teuchis* and 46% for *G*. *salaris* and *G*. *thymalli*) and the transition/transversion ratio (5.98 for *G*. *teuchis* and 1.1 for *G*. *salaris* and *G*. *thymalli*) differ significantly. It remains thus open to what extent the differences in the estimated substitution rates reflect (i) lineage differences, (ii) sampling bias, and/or (iii) uncertainties in the assumptions that the estimates rest on. Nevertheless, the substitution rate determined here for the mitochondrial COI gene of *G*. *teuchis* is very close to the estimate of 5.5% suggested for the ITS region of the nuclear rDNA cluster of *Gyrodactylus*, an estimate derived from vicariance of *G*. *aphyae* on the Eurasian minnow (*Phoxinus phoxinus*) on opposite sides of the Baltic–White Sea watershed [15]. This seems surprising as mitochondrial DNA in animals generally evolves substantially faster than nuclear loci, but the variation among taxonomic groups is large, and for some basal groups (e.g. corals) the opposite is true (see [55,56], and references therein). Huyse & Volckaert [18] reported the ITS region to evolve at a rate of only 0.07% per My in *G*. *branchialis*, an estimate in the same order of magnitude as the low genetic diversity of the ITS2 region observed in *G*. *teuchis*.

The current study not only suggests co-speciation of *G*. *teuchis* and its salmonids hosts, but also documents pronounced differences in the phylogeographic structure and the evolution of the host parasite systems between *G*. *teuchis* and *G*. *truttae*. While the association of *G*. *teuchis* with its host lineages seems to result from co-divergence, the relatively low genetic diversity and the lack of phylogeographic structure in *G*. *truttae* might indicate a very recent host switch onto brown trout (or a historical bottleneck) and a subsequent rapid range expansion; however, based on the limited sampling, the host-parasite association of *G*. *truttae* and *Salmo* spp. appears considerably more recent than for *G*. *teuchis*. Human mediated transfer of trout stocks may have significantly enhanced the spread of *G*. *truttae*. Applying the nucleotide substitution rate determined for COI of *G*. *teuchis*, the observed genetic diversity implies an age of <60,000 years for the most recent common ancestor of all currently identified *G*. *truttae* haplotypes, significantly younger than the splits among major mtDNA lineages of *S*. *trutta*.

### Taxonomic implications

Species delimitation in *Gyrodactylus* is a difficult issue. Morphological species identification is usually based on only a few characters and is far from straightforward as it requires sophisticated morphometric techniques [57]. Molecular species identification in *Gyrodactylus* was therefore highly welcomed, and currently relies largely on sequencing of the ribosomal internal transcribed spacers (ITS). However, the current study reveals substantial difference in genetic diversity between ITS and the mtDNA COI. The PTP algorithm splits *G*. *teuchis* into two to five putative species based on COI data while the ITS data are in favour of *G*. *teuchis* being only one species. It is particularly interesting that the COI haplotypes of clade E, which were the only ones detected in parasites infecting Atlantic salmon differ from all other haplotypes by roughly 10%. Neither the original description of *G*. *teuchis* [58] nor the re-descriptions [29,45] were accompanied by COI sequences and genetic information was until now restricted to the ITS sequences produced by these studies. However, the river Scorff, sampled in the current study, was one of the localities where *G*. *teuchis* had initially been reported [58]. The COI data obtained for the river Kleiner Kamp in the current study is based on the same material that was used in the re-description of *G*. *teuchis* [29].

A similar pattern of differential resolution between ITS and COI sequence diversity has been reported for the notorious salmon parasite *G*. *salaris* and its benign sibling species *G*. *thymalli* infecting European grayling (*T*. *thymallus*). Mitochondrial COI haplogroups in *G*. *salaris* and *G*. *thymalli* differ by ~2–3% while the ribosomal ITS sequences are identical [15], reflecting a considerably lower substitution rate. The ITS data were taken as an indication of conspecificity by some authors [15] (see also [59] for review), while others reject this conclusion based on experimental evidence showing differences in host specificity and possibly co-evolutionary adaptations to the respective hosts [60,61]. In a recent study, Fromm et al. [62] investigated microRNA loci in *G*. *salaris* and *G*. *thymalli* and detected very low sequence diversity, which was interpreted as further support for the hypothesis of conspecificity initially drawn from the ITS data. However, in *G*. *thymalli* the various mitochondrial haplogroups seem to largely correspond to different European drainage systems [63]. The sister species *G*. *salmonis* and *G*. *salvelini* infecting North American *Salvelinus fontinalis* and European *S*. *alpinus*, respectively, represent a further example of a taxonomic dilemma. While the species show a > 2.5% mitochondrial divergence [64] they likewise share identical ITS sequences.

The current study emphasizes once more that taxonomic conclusions based on single molecular markers can be ambiguous. The application of high throughput genome-wide analyses will certainly add a new dimension to understanding the short-term evolutionary history in *Gyrodactylus* species and is likely to challenge our current view of species boundaries in this highly diverse, yet relatively understudied genus. However, we adhere to a view that molecular distances alone should not be used to revise taxonomy. Nonetheless, the molecular divergence for clade E within the *G*. *teuchis* complex is approximately 10% and far exceeds distances typically associated with species delineation, and further, this clade is associated with a shift in the host species, altogether supporting the main thesis that our study demonstrates co-speciation. However, this is not a taxonomic study, and in the absence of morphological data and experimental evaluations of potential reproductive barriers we render a taxonomic revision of the *G*. *teuchis* complex premature. Once more data is available, *G*. *teuchis* may become an ideal model species for exploring fine scale host-parasite interactions of monogenean fish parasites.
